# Early dysregulation of the memory CD8^+^ T cell repertoire leads to compromised immune responses to secondary viral infection in the aged

**DOI:** 10.1186/1742-4933-9-28

**Published:** 2012-12-18

**Authors:** Lisa M Connor, Jacob E Kohlmeier, Lynn Ryan, Alan D Roberts, Tres Cookenham, Marcia A Blackman, David L Woodland

**Affiliations:** 1 Trudeau Institute, Saranac Lake, NY, USA

**Keywords:** T cell receptor repertoire, Acute respiratory virus infection, CD8^+^ T cell memory maintenance, T cell clonal expansion

## Abstract

**Background:**

Virus-specific memory CD8^+^ T cells persist long after infection is resolved and are important for mediating recall responses to secondary infection. Although the number of memory T cells remains relatively constant over time, little is known about the overall stability of the memory T cell pool, particularly with respect to T cell clonal diversity. In this study we developed a novel assay to measure the composition of the memory T cell pool in large cohorts of mice over time following respiratory virus infection.

**Results:**

We find that the clonal composition of the virus-specific memory CD8^+^ T cell pool begins to change within months of the initial infection. These early clonal perturbations eventually result in large clonal expansions that have been associated with ageing.

**Conclusions:**

Maintenance of clonal diversity is important for effective long-term memory responses and dysregulation of the memory response begins early after infection.

## Background

Acute respiratory viral infection results in the generation of memory CD8^+^ T cells that persist in high frequencies for years after antigen clearance [1-3]. These memory CD8^+^ T cells mediate accelerated viral clearance following secondary infection and can result in protection from death under certain circumstances [4-6]. Given the importance of memory CD8^+^ T cells in protective immunity, understanding the maintenance of this population is important for vaccine development.

Memory T cell numbers remain relatively constant for many years post-infection. This is largely a consequence of homeostatic turnover driven by the cytokines IL-15 and IL-7 [7-9], without requiring the presence of antigen or MHC molecules [10,11]. However, it is unclear whether homeostatic proliferation is sufficient to maintain the clonal diversity of the memory T cell pool. Previous studies have shown that CD8^+^ memory T cell pools can become dysregulated over time, culminating in the appearance of large monoclonal expansions of T cells (TCE) in aged humans and mice [12-17]. Adoptive transfer experiments and BrdU incorporation demonstrate that large TCE exhibit higher rates of homeostatic proliferation, which is likely to contribute to the development of these expansions. In mouse models of Sendai or influenza virus infection, we recently demonstrated that, in extreme cases, virus-specific CD8^+^ memory T cells could comprise upwards of 90% of the entire CD8^+^ T cell pool by 22 months post-infection. These virus-specific T cell expansions are usually first identified when they comprise greater than 10% of the entire CD8^+^ T cell pool, typically around 15 months post-infection. Interestingly, although clonally expanded memory T cells are present in elevated numbers and maintain effective cytokine and cytotoxic activity ex vivo, most of these expanded clones are significantly impaired in their capacity to mount recall responses to secondary challenge in vivo [15].

Current methods to detect clonal expansions are insensitive to small changes in the memory T cell repertoire and are only able to identify the extreme examples of memory T cell dysregulation (when the clonal expansion represents greater than 10% of the CD8^+^ T cell population). Based on the insensitivity of the standard assay, we hypothesized that we may be greatly underestimating when the T cell repertoire first begins to degrade. To address this, we developed a sensitive assay to measure the composition of the memory T cell pool following acute respiratory virus infection. Using this assay, we show that perturbations occur in the memory T cell pool well before the appearance of large clonal expansions, in a substantial proportion of animals. In addition, these small perturbations exhibit similar functional abnormalities that have been observed with large clonal expansions; including impaired ability to respond to secondary infection and altered homeostatic proliferation.

## Results and discussion

### Small perturbations occur in the virus specific memory T cell pool and give rise to large TCE

**Figure 1:**
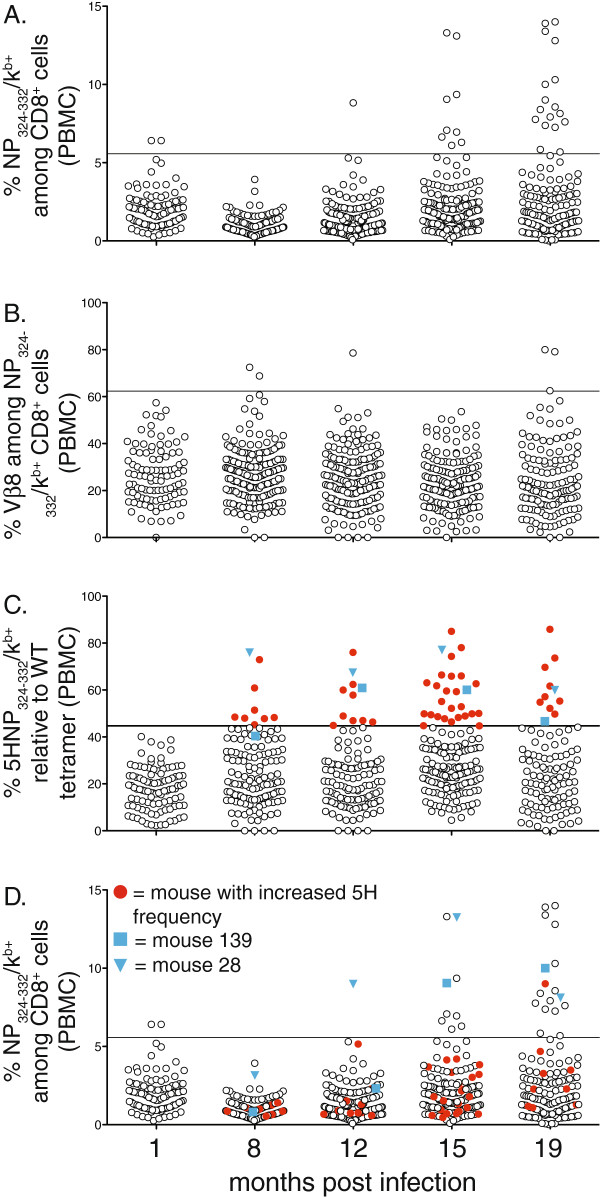
**Progressive dysregulation of the virus-specific CD8**^**+ **^**T cell repertoire over time.** Cohorts of 200 and 100 B6 mice were infected with Sendai virus and bled at 1 (100 mouse cohort), 8, 12, 15 and 19 (200 mouse cohort) months post infection. **A**, The frequency of NP_324-332_K^b+^ cells among total CD8^+^ T cells and B, frequency of Vβ8^+^ cells among NP_324-332_K^b+^ cells for each individual mouse is graphed over time. **C**, The percentage of 5HNP_324-332_K^b+^ cells relative to NP_324-332_K^b+^ cells is shown, filled red circles indicate mice with frequencies greater than 3SDs from the normal frequency observed at 1 month (horizontal line). **D**, The frequency of NP_324-332_K^b+^ cells among total CD8^+^ T cells is shown for mice with increased 5H and are indicated by red filled circles. In all panels, horizontal line indicates frequency greater than 3SDs from the normal frequency observed at 1 month. In panels C and D, blue squares and triangles represent mouse 139 and 28 respectively.

We infected large cohorts of C57BL/6 mice with Sendai virus and tracked the pool of memory CD8^+^ T cells specific for the immunodominant NP_324-332_/K^b^ epitope. Blood samples were taken from mice at various times post-infection and the frequency of NP_324-332_/K^b^-specific T cells among total CD8^+^ T cells was determined. As reported previously, the frequency of NP_324-332_/K^b^-specific T cells at one month post infection averaged 2% with a range of 0.3-6.4% (Figure 1A). Using the one month time point as the standard, it was apparent that large clonal expansions (ie greater than 3SD above the average frequency at one month post-infection) began to appear in several mice by 15 months and were present in 8.5% of mice by 19 months. In an attempt to detect smaller perturbations in the NP_324-332_/K^b^-specific T cell repertoire, we examined TCR Vβ8 usage. Our idea was that perturbations in the antigen-specific T cell pool would be detected by a shift in TCR Vβ usage, even in the absence of a significant increase in the overall frequency of cells. However, as shown in Figure 1B, the range of Vβ8 usage was very large at one month post infection, making it difficult to identify significant changes over time.

**Figure 2:**
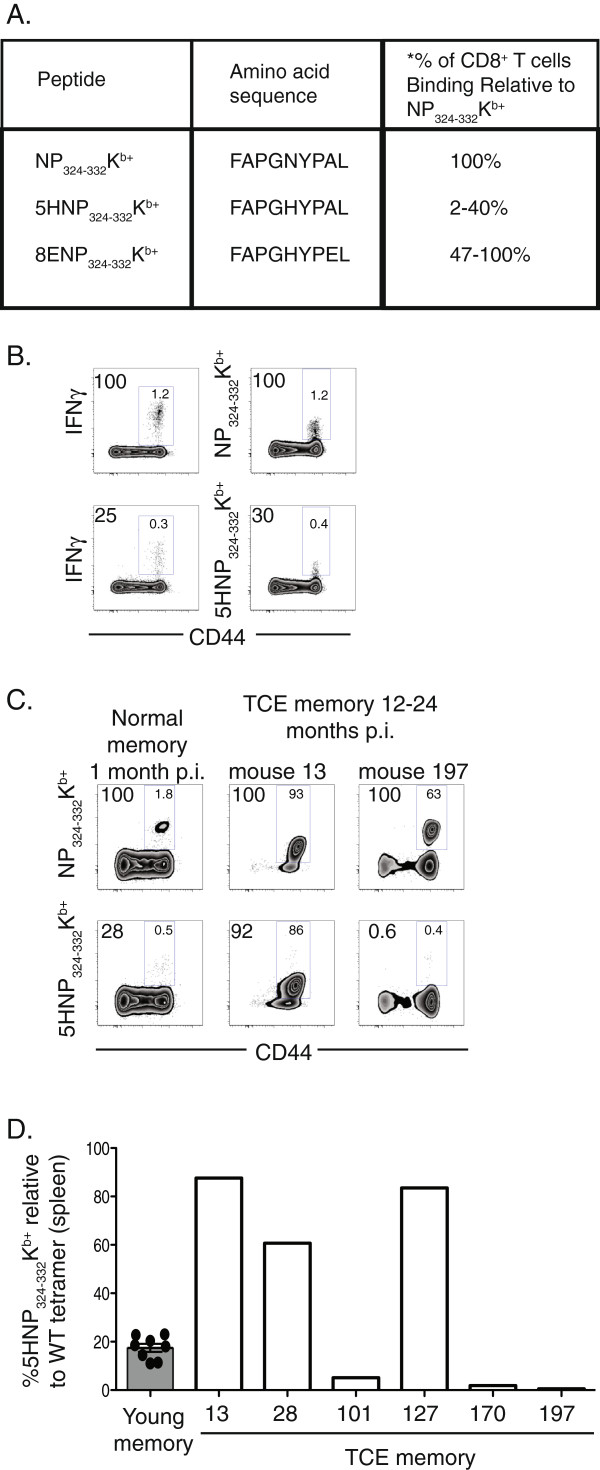
**Detecting perturbations in the memory T cell pool using an altered peptide approach.** NP_324-332_K^b+^ and altered peptide amino acid sequences are shown in **A**. Spleen cells from mice infected with Sendai virus 1 month earlier were stimulated with 1μg of either NP_324-332_K^b^ (B, top left) or 5HNP_324-332_K^b^ (**B**, bottom left) peptide in the presence of BFA and IL-2 or were stained with NP_324-332_K^b+^ or 5HNP_324-332_K^b+^ tetramers, CD8 and CD44. Flow cytometric plots show intracellular IFNγ production (B, left) or frequency of tetramer (B, right) in CD8 gated T cells. **C**, Peripheral blood from mice with normal (1 month post infection) (left) and TCE (12–24 month post infection, mouse #s 13 and 197) (middle and right) memory CD8^+^ T cell pools. Flow cytometric plots show frequency of NP_324-332_K^b+^- (top) and 5HNP_324-332_K^b+^- (bottom) specific cells among CD8^+^ T cells. Numbers in the gate indicate frequency of cells among CD8^+^ T cells, numbers outside the gate represent frequency of cells relative to frequency of NP_324-332_K^b+^ cells. **D**, Data show frequency of 5HNP_324-332_K^b+^ relative to NP_324-332_K^b+^ cells from 1 month memory mice, (symbols indicate individual mice and bar represents mean value) and of 6 individual memory mice 12–24 months post infection exhibiting TCE (>20% NP_324-332_K^b+^cells). * Represents range % of CD8^+^ T cells binding to tetramer relative to NP_324-332_K^b+^ based on blood samples taken from 200 Sendai virus infected mice.

As an alternative approach to studying repertoire perturbations, we took advantage of altered NP_324-332_/K^b^ peptides to subdivide the memory T cell pool. Previous studies had shown that subsets of T cells specific for the NP_324-332_/K^b^ epitope are able to recognize altered peptides (in which T cell receptor contact residues had been replaced without affecting MHC binding) bound to K^b^ (Figure 2A) [18]. We focused on one peptide that had an amino acid substitution at position 5 (replacing Asparagine with Histidine, which we have denoted as 5HNP_324-332_/K^b^). This altered peptide stimulates IFNγ production in approximately 25-30% of the total NP_324-332_/K^b+^ T cell pool elicited by Sendai virus infection (Figure 2B). Tetramer reagents prepared with the 5HNP_324-332_ peptide binds to on average 25-30% of the total NP_324-332_/K^b^-specific T cell memory pool from mice 1 month post-infection. Importantly, the fraction of the memory T cell pool binding to 5HNP_324-332_/K^b^ was relatively conserved in the young memory T cell pool (range of 2-40%, Figure 1C). To confirm that the altered peptide tetramers would detect perturbations in the repertoire of NP_324-332_/K^b^-specific memory T cells we stained peripheral blood from mice with large clonal expansions based on the overall frequency of NP_324-332_/K^b^-specific T cells (>10% of total CD8^+^ T cell pool). As shown in Figure 2C and D, the fraction of NP_324-332_/K^b^-specific memory T cells that bound the 5HNP_324-332_/K^b^ tetramer was either markedly increased or almost completely absent, consistent with a reduction in the overall clonality of the population. Together, these results suggest that analysis of altered peptides can be used to identify changes in the diversity of the memory T cell repertoire.

We next used the altered peptide approach to study changes in the repertoire of the memory T cell pool over time. Using blood samples from the same cohorts of Sendai virus infected mice described in Figure 1A, we determined the fraction of the antigen-specific memory T cell pool that bound the 5HNP_324-332_/K^b^ tetramer. Perturbations in the memory T cell pools of individual mice were defined by altered tetramer binding that was 3SD above the normal range of young (1 month) memory. Following this stringent criteria, we detected significant perturbations in the memory T cell pool in >4% of mice as early as 8 months post infection (Figure 1C, filled red circles). Consistent with our hypothesis, many of the mice exhibiting large repertoire perturbation based on the altered peptide approach did not exhibit an overall increase in the frequency of NP_324-332_/K^b^-specific T cells.

To illustrate that altered peptides allow us to detect changes in the repertoire even when there is no change in frequency of NP_324-332_/K^b+^ cells we tracked the frequencies of 5HNP_324-332_/K^b+^ and NP_324-332_/K^b+^ cells from individual mice over time. For example, mouse 139 (blue squares) and 28 (blue triangles) have significant repertoire perturbations even though they expressed normal NP_324-332_/K^b^-specific T cell frequencies at 8 months post infection (Figure 1D), however, the frequency of NP_324-332_/K^b^-specific cells gradually increased over time and both animals exhibited expansions in the CD8^+^ T cell pool by 15 months post infection (≥ 10% NP_324-332_/K^b^-specific cells among CD8^+^ T cells). In both cases, mice expressed elevated levels of 5HNP_324-332_/K^b^-specific T cells within the memory T cell pool, which could be detected as early as 8 months post infection (Figure 1C).

It should be noted that this assay underestimates the clonal dysregulation since the assay only considers on average 25-30% of the T cell clones in the normal antigen-specific memory T cell repertoire (note that on average 70-75% of T cell clones fail to recognize 5HNP_324-332_/K^b^ at 1 month-post infection). Therefore, if we extrapolate these data to include the remaining 70-75% of T cell clones, the frequency of individuals that exhibit repertoire perturbations is likely to be 3–4 fold greater than what we can observe with the 5HNP_324-332_/K^b^ tetramer.

**Figure 3:**
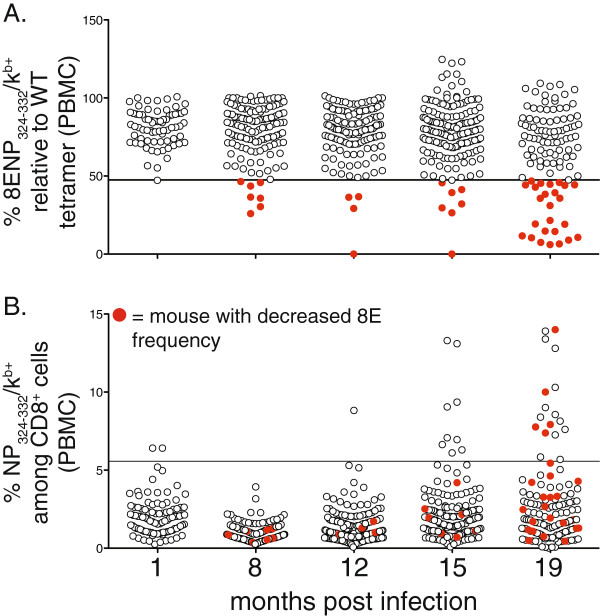
**Progressive dysregulation of the virus-specific CD8**^**+ **^**T cell repertoire over time.** Using the same cohorts of mice described in Figure 1, blood was collected from individual mice at 1, 8, 12, 15 and 19 months post infection and stained with either NP_324-332_K^b^ tetramer or altered peptide tetramer, 8ENP_324-332_K^b^ and analyzed by flow cytometry. **A**, The percentage of 8ENP_324-332_K^b+^ cells relative to NP_324-332_K^b+^ cells is shown, filled red circles indicate mice with frequencies less than 3SDs from the normal frequency observed at 1 month (horizontal line). **B**, The frequency of NP_324-332_K^b+^ cells among total CD8^+^ T cells are shown for mice with decreased 8E and are indicated by red filled circles. In all panels, the horizontal line indicates frequency greater than 3SDs from the normal frequency observed at 1 month.

One possible concern with these data is that T cells specific for 5HNP_324-332_/K^b^ might be more unstable than the general population and preferentially become perturbed. Therefore, we examined the frequency of the antigen-specific T cell pool for a different altered peptide, 8ENP_324-332_/K^b^. This altered peptide had an amino acid substitution at position 8 (replacing alanine with glutamic acid), and was recognized by on average 80% of NP_324-332_/K^b^-specific T cells (Figure 2A). In this case, perturbations in the antigen-specific T cell pool could be identified by the loss of 8ENP_324-332_/K^b^ tetramer binding. As shown in Figure 3A**,** the number of individual mice exhibiting perturbed 8ENP_324-332_/K^b^ frequencies (< 47% 8ENP_324-332_/K^b^-specific T cells relative to total NP_324-332_/K^b+^ cells) was similar to the data obtained using 5HNP_324-332_/K^b^ tetramer. Furthermore, perturbations could be detected as early as 8 months post infection even though the frequency of total NP_324-332_/K^b^-specific cells in affected mice was within the normal range (Figure 3B). Of importance, in most cases, individual mice exhibiting perturbed 8ENP_324-332_/K^b^ frequencies were not the same mice with 5HNP_324-332_/K^b^ perturbations (Figures 1D and 3B). This supports our hypothesis that the data obtained using one altered peptide underestimates the total frequency of mice that exhibit repertoire perturbations.

Together, the data confirm the findings with the 5HNP_324-332_/K^b^ tetramer, that smaller perturbations within the memory T cell pool appear earlier and occur in a significantly greater proportion of animals than previously recognized when measuring total frequency of NP_324-332_/K^b^-specific T cells among the CD8^+^ T cell population.

### Small perturbations in the memory T cell pool exhibit dysfunctional immune responses

**Figure 4:**
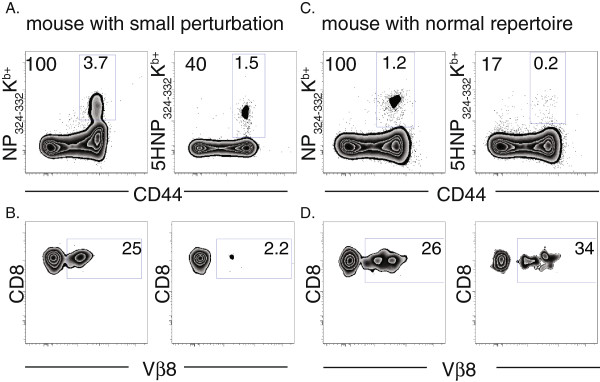
**Mice with 5HNP**_**324-332**_**/K**^**b **^**perturbations exhibit memory T cell populations that are less clonally diverse.** Representative flow cytometric plots from Sendai virus infected mice (<1yr post infection) with a small perturbation (**A** and **B**) or normal memory T cell repertoire (**C** and **D**). Frequency of NP_324-332_K^b+^ (**A** and **C**, left plot), 5HNP_324-332_K^b+^ (**A** and **C**, right plot) and their Vβ8 usage (**B** and **D**) are shown. Plots are gated on CD8^+^ (**A** and **C**) or tetramer^+^ (**B** and **D**) populations. Numbers in the gate indicate frequency of cells among CD8^+^ T cells, numbers outside the gate represent frequency of cells relative to frequency of NP_324-332_K^b+^ cells.

We next asked whether small perturbations within the memory T cell pool resulted in dysfunctional immune responsiveness. To address this question, we first identified mice exhibiting a marked increase in the fraction of memory T cells recognizing 5HNP_324-332_/K^b^ (>40% of memory T cells), consistent with a clonal expansion within the memory T cell pool. Lymphocytes isolated from these mice were stained with the WT NP_324-332_/K^b^ tetramer and the 5HNP_324-332_/K^b^ tetramer, allowing us to discriminate between the perturbed memory T cells (within the 5HNP_324-332_/K^b^ positive population) from the rest of the memory T cell pool (NP_324-332_/K^b^ positive; 5HNP_324-332_/K^b^ negative). An example is shown in Figure 4A where a perturbation in the memory T cell pool was identified based on the elevated frequency of cells binding to 5HNP_324-332_/K^b^ tetramer relative to the total frequency of the antigen specific pool in the blood. In contrast, the fraction of the total memory pool binding to 5HNP_324-332_/K^b^ tetramer in an aged matched individual with a normal T cell repertoire was within the normal range of young (1 month) memory (Figure 4C) The perturbation illustrated in Figure 4C was further highlighted by examining Vβ8 usage between groups (Figure 4B and D). In mice with a small 5HNP_324-332_/K^b^ perturbation, we found that the frequency of Vβ8^+^ T cells in 5HNP_324-332_/K^b^-specific population was markedly decreased (Figure 4B, right plot), further supporting our claim that cells binding to the 5HNP_324-332_/K^b^ tetramer are significantly less clonally diverse in mice with 5HNP_324-332_/K^b^ perturbations. In contrast, the frequency of Vβ8^+^ T cells in the NP_324-332_/K^b^-specific population was similar to an individual with a normal memory T cell repertoire (Figure 4B, left plot).

**Figure 5:**
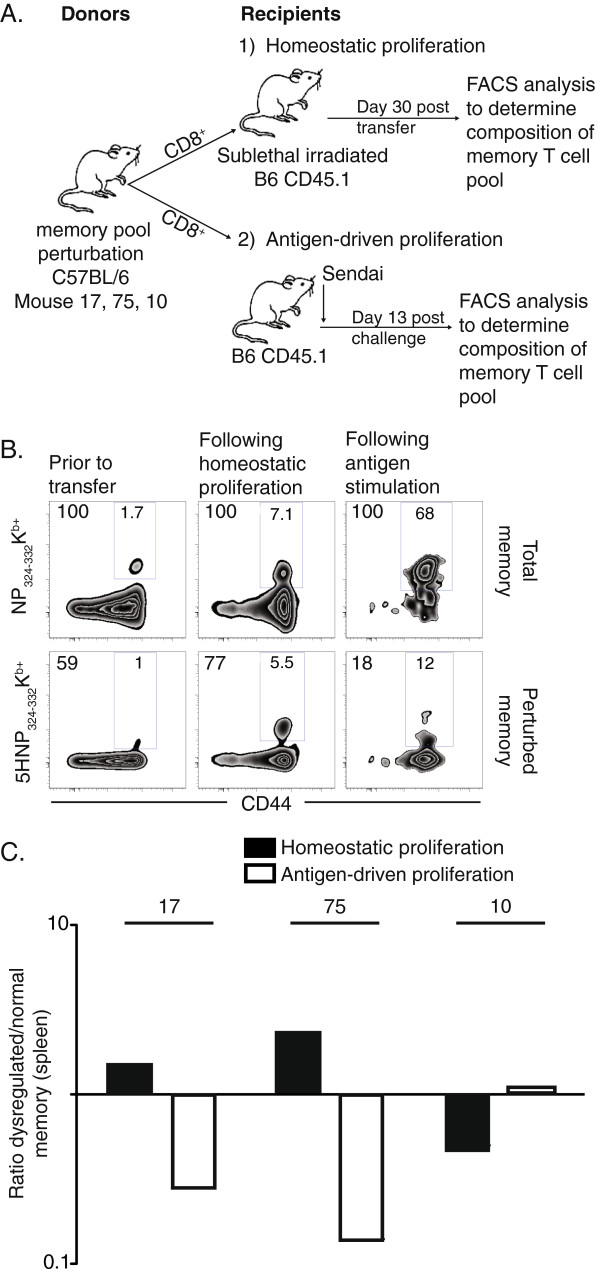
**Small perturbations in the memory T cell pool exhibit dysfunctional immune responses.A**, CD8^+^ T cells isolated from mice with 5HNP_324-332_K^b+^ perturbations (%5HNP_324-332_K^b+^ relative to NP_324-332_K^b+^: mouse 17, 60%; mouse 75, 59%; mouse 10, 45%) were isolated and used in adoptive transfer experiments depicted by the diagram to measure homeostatic proliferation (top) and antigen-driven proliferation (bottom). A detailed description of the protocol can be found in the methods section. **B**, Representative staining of NP_324-332_K^b+^ and 5HNP_324-332_K^b+^ populations from spleen of mouse 75 is shown gated on CD45.2^+^ CD8^+^ cells; numbers inside the gate indicate frequency of tetramer positive cells among CD8^+^ T cells, numbers at upper left corner of plots represent frequency of cells relative to frequency of NP_324-332_K^b+^ cells (% tetramer frequency ÷ % NP_324-332_K^b+^) x100). **C**, Data from adoptive transfer experiments from three different donors (mouse 17, 75 and 10) is graphed as the ratio of perturbed (%5HNP_324-332_K^b+^) to the rest of the memory T cells (%NP_324-332_K^b+^ − %5HNP_324-332_K^b+^) from the spleen. Ratios for each population were normalized to the Ratio prior to transfer. Number of recipients ranged from 1 to 2 for each donor mouse.

We next assessed the ability of perturbed memory T cell populations to proliferate either homeostatically or to cognate antigen (Figure 5A). Spleen cells from three mice (mouse 17, 75 and 10) bearing small perturbations (>40% 5HNP_324-332_/K^b^ among antigen-specific cells) were enriched for CD8^+^ T cells and the ratio of perturbed to normal memory T cells was determined by tetramer staining (Mouse #75, Figure 5B, left panels). First, cells were transferred into sublethally irradiated B6 CD45.1 recipients and the rate of homeostatic proliferation of perturbed memory T cells was determined by comparing the ratio of normal to perturbed memory T cells prior to and 30 days after transfer using tetramer staining (Mouse #75, Figure 5B middle panels, homeostatic proliferation). The ratio of normal to perturbed memory is shown in Figure 5C for all three mice (closed bars). It can be seen that perturbed memory T cells from mouse 17 and 75 had a higher rate of homeostatic proliferation compared to the rest of the memory T cells (1.5 and 2.3 fold greater expansion, respectively). In contrast, perturbed memory T cells isolated from mouse 10 proliferated less. These data suggest that perturbed memory T cells vary in their rate of homeostatic proliferation, relative to the rest of the memory T cell pool. Second, to assess the ability of perturbed memory T cells to proliferate in response to antigen stimulation, CD8^+^ T cells isolated from mice with small perturbations (mouse 17, 75 and 10) were transferred into B6 CD45.1 recipients 1 day prior to Sendai virus infection and the ratio of normal to perturbed memory T cells before transfer was compared to the ratio at 13 days post infection (Figure 5A, Antigen-driven proliferation). The tetramer staining is shown in Figure 5B for mouse #75 (right panels) and the ratio for all three mice is plotted in Figure 5C (open bars). The data show that perturbed memory T cells from mouse 17 and 75 proliferated 3.5 and 7 fold less in response to antigen stimulation than unperturbed memory T cells. In contrast, antigen-driven proliferation by perturbed memory T cells isolated from mouse 10 was comparable to unperturbed memory (Figure 5C). As this population of memory T cells also exhibited a normal rate of cell division under lymphopenic conditions, the data show that, in some cases, perturbed memory T cells can retain immune responsiveness.

Large TCE are more frequently detected as individuals age, leading to the hypothesis that these expansions are triggered by changes in the aged environment. However, here we have used a sensitive assay to show that the composition of the memory T cell pool undergoes significant alterations as early as 8 months post infection and occurs in a significant proportion of animals.

The extent to which perturbations in the memory T cell pool becomes detrimental for host immunity to secondary infection is not clear. Previous studies using TCE of unknown specificity show that their presence within the CD8^+^ T cell pool significantly limits the availability of T cells capable of responding to new infections, resulting in increased susceptibility to infection [19,20]. In this study we demonstrate that the majority of perturbed T cells are unable to participate in recall responses. By contrast, the residual ‘normal’ memory T cells mounted robust response to antigen. Together these data suggest that perturbations in the memory T cell pool have a negative impact on the total memory T cell pool progressively limiting the number of memory T cells that are capable of responding to a secondary infection as they increase in size. Of note, not all perturbed memory T cells have impaired recall responses. In some cases, perturbed immunity mounted equivalent responses to normal memory T cells. This suggests the degradation of the memory pool is highly stochastic, and although in most cases perturbed responses are detrimental to host immunity, sometimes they can participate in the recall response. In summary, the presence of a TCE can significantly limit the availability of T cells capable of responding to pathogens they are specific for and unrelated pathogens [19,20]. Therefore, TCE have a negative impact on immune responses to new and previously encountered pathogens, resulting in an increase in susceptibility to infection.

The long-term maintenance of memory T cells relies on continuous homeostatic proliferation. The data from this study suggest that renewal of memory CD8^+^ T cells by homeostatic proliferation maintains T cell numbers but may not be adequate for maintaining repertoire diversity or function of memory CD8^+^ T cells over time. One possible explanation is that perturbations arise as a result of stochastic, asynchronous cell division. In this case, one T cell clone maintains a slightly higher division rate than another clone, resulting in the gradual expansion of dominant T cell clones in the CD8^+^ T cell pool. In support of this hypothesis, we showed that perturbed memory T cells isolated from two of three mice expressing perturbations in the virus-specific memory T cell pool proliferate more than normal memory when transferred into lymphopenic hosts. In addition, studies using adoptive transfer approaches or BrdU incorporation have shown that clonally expanded memory CD8^+^ T cells from aged mice undergo more cell division compared to normal memory populations [12,15,21]. Thus, the increased rate of homeostatic turnover by individual CD8^+^ T cell clones can contribute to the development of memory T cell perturbations.

The fact that perturbed memory T cells frequently failed to respond to secondary antigen challenge is likely to have a negative impact on the efficacy of the recall response. However, it should be noted that secondary challenge skewed the response in favor of normal memory T cells by purging perturbed memory CD8^+^ T cells from the population. Thus, enriching for responsive T cells may restore the memory T cell pool efficacy. With this in mind, it may be important to readdress whether cognate antigen is important for maintaining long-term memory CD8^+^ T cells. Evidence that MHC class I interactions are not required for long-term maintenance of memory T cells is based on the ability of memory T cells to divide by homeostatic proliferation and maintain elevated precursor frequencies in the absence of MHC class I [10,11]. In addition, cytokine production following in vitro restimulation with cognate antigen suggests the functional capacity of these memory CD8^+^ T cells is intact. Importantly, we have shown that memory T cell pools exhibiting substantial perturbation mediate a reduced capacity to participate in a recall response, despite being present in large numbers and retaining the capacity to secrete IFNγ and TNFα ex vivo [15]. Therefore, it is possible that booster vaccinations could be important for restoring effective memory T cell populations and promoting long-term maintenance.

## Conclusions

The findings from this study suggest that the maintenance of the long-term memory T cell pool generated following an acute viral infection deteriorates over time, with perturbations beginning as early as 8 months post infection. These perturbations can lead to a substantially impaired capacity to mount recall responses. Importantly we demonstrated the potential for antigen restimulation to restore immune responsive memory T cells. Thus, these results have important implications for vaccine design, particularly with respect to booster vaccination regimes that could overcome the increasingly dysregulated immune response in the elderly.

## Materials and methods

### Mice, viruses, and infections

C57BL/6 and B6.SJL-*Ptprca* Pep3/BoyJ (CD45.1) mice were purchased from The Jackson Laboratory and re-derived stocks were maintained at the Trudeau Institute. Sendai virus (Enders strain) was grown, stored, and titered as previously described [22]. To facilitate intranasal infections, mice were anesthetized with 2,2,2-tribromoethanol (200mg/kg) and virus was administered in a volume of 30μL. For analysis of long-term memory T cell responses, two cohorts of 200 and 100 6–8 week old C57BL/6 mice were intranasally infected with 250 50% egg infectious doses (EID_50_) of Sendai virus. All animal studies were approved by the Trudeau Institute Animal Care and Use Committee.

### Tissue harvest

For serial bleeds, peripheral blood (approximately 100μl) was obtained by nicking the tail vein and diluted 1:2 in PBS containing 10U/ml heparin. For endpoint assays, cells were isolated from the spleen by mechanical disruption. Following red blood cell lysis with ammonium buffered chloride, live cell numbers were determined by counting and trypan blue exclusion.

### Flow cytometry

Single cell suspensions were incubated with Fc-block (anti-CD16/32) for 15 minutes on ice followed by staining with tetramer reagents (SenNP_324-332_K^b^, 5HNP_324-332_K^b^, 8ENP_324-332_K^b^) for 1 hour at room temperature. Tetramers were generated by the Trudeau Institute Molecular Biology Core. Tetramer-labeled cells were incubated with antibodies to surface proteins for 30 minutes on ice. Antibodies were purchased from BD Biosciences (All TCR Vβ antibodies, CD45.2) and eBioscience (CD8, CD44). Samples were run on a FACS Canto II flow cytometer (BD Biosciences) and data were analyzed with Flow Jo software (TreeStar).

### CD8 T cell enrichment and adoptive transfer

For CD8^+^ T cell enrichment, total splenocytes were stained with Biotin Mouse CD8 T Lymphocyte Enrichment Cocktail (BD Biosciences) per manufacture’s instructions and CD8^+^ T cells were negatively selected using magnetic beads. An aliquot of enriched cells was stained with NP_324-332_K^b^ and 5HNP_324-332_K^b^ tetramer as described above to determine the number of Sendai-specific CD8^+^ T cells. To assess responses to antigen, 1×10^4^ Sendai-specific CD8^+^ T cells were intravenously transferred into naive B6 CD45.1 recipients and then intranasally challenged with 250 EID_50_ of Sendai virus one day later. On day 13 post-infection, lymphocytes were isolated from various tissues and host and donor Sendai-specific cells were identified by flow cytometry. The relative response in each tissue was calculated from the frequency of Sendai NP_324–332_/K^b^- and 5HNP_324-332_K^b^-specific T cells. To assess homeostatic proliferation, 5×10^4^ Sendai-specific CD8^+^ T cells were intravenously transferred into naïve B6 CD45.1 recipients that were exposed to 600 cGy whole body irradiation. On day 30 post transfer, lymphocytes were isolated and the relative responses were determined as described above.

### Intracellular cytokine staining

For measurement of cytokine production, single cell suspensions were incubated with NP_324-332_/K^b^, 5HNP_324-332_K^b^ or control peptides as previously described [23]. Cells were stained for surface markers, fixed and permeabilized (CytoFix/CytoPerm kit, BD Biosciences), and stained for intracellular cytokines with antibodies to IFN-γ (BD biosciences).

### Statistics

Statistical analysis was performed with Prism GraphPad software, and significance was determined by an unpaired two-tailed Student’s *t* test. P-values less than 0.05 was considered significant.

## Abbreviations

TCE:T cell clonal expansions; TCR: T cell receptor.

## Competing interests

The authors declare that they have no competing interests.

## Authors’ contributions

LC, JK, MB and DW designed the study, contributed to interpretation of the data and wrote the manuscript; LC analyzed the data; LC and LR developed and tested altered peptides. LC, LR, TC and AR performed serial bleeds and flow cytometry; LC and LR performed the adoptive transfer studies. All authors read and approved the final manuscript.
