# Correction: Early dysregulation of the memory CD8^+^ T cell repertoire leads to compromised immune responses to secondary viral infection in the aged

**DOI:** 10.1186/1742-4933-10-40

**Published:** 2013-10-11

**Authors:** Lisa M Connor, Jacob E Kohlmeier, Lynn Ryan, Alan D Roberts, Tres Cookenham, Adam Quinn, Marcia A Blackman, David L Woodland

**Affiliations:** 1 Trudeau Institute, Saranac Lake, NY, USA

## Correction

After publication of this work [1], we noted that Adam Quinn was inadvertently left off of the author list. The full list of authors has now been added and the Authors’ contributions and the Competing interest section have been appropriately modified.

We also failed to acknowledge the funding sources that supported this work, which we have added to the acknowledgements section.

## Competing interests

The authors declare that they have no competing interests.

## Authors’ contributions

LC, JK, MB and DW designed the study, contributed to interpretation of the data and wrote the manuscript; LC analyzed the data; LC and LR developed and tested altered peptides; LC, LR, TC, AR and AQ performed serial bleeds and flow cytometry; LC and LR performed the adoptive transfer studies. All authors read and approved the final manuscript.
